# Distributed computing in image analysis using open source frameworks and application to image sharpness assessment of histological whole slide images

**DOI:** 10.1186/1746-1596-6-S1-S16

**Published:** 2011-03-30

**Authors:** Norman Zerbe, Peter Hufnagl, Karsten Schlüns

**Affiliations:** 1 Institute of Pathology, Charité – Universitätsmedizin Berlin, Charitéplatz 1, 10117, Berlin, Germany; 2 University of Applied Sciences Berlin, Wilhelminenhofstraße 75A, 12459, Berlin, Germany

## Abstract

**Background:**

Automated image analysis on virtual slides is evolving rapidly and will play an important role in the future of digital pathology. Due to the image size, the computational cost of processing whole slide images (WSIs) in full resolution is immense. Moreover, image analysis requires well focused images in high magnification.

**Methods:**

We present a system that merges virtual microscopy techniques, open source image analysis software, and distributed parallel processing. We have integrated the parallel processing framework JPPF, so batch processing can be performed distributed and in parallel. All resulting meta data and image data are collected and merged. As an example the system is applied to the specific task of image sharpness assessment. ImageJ is an open source image editing and processing framework developed at the NIH having a large user community that contributes image processing algorithms wrapped as plug-ins in a wide field of life science applications. We developed an ImageJ plug-in that supports both basic interactive virtual microscope and batch processing functionality. For the application of sharpness inspection we employ an approach with non-overlapping tiles. Compute nodes retrieve image tiles of moderate size from the streaming server and compute the focus measure. Each tile is divided into small sub images to calculate an edge based sharpness criterion which is used for classification. The results are aggregated in a sharpness map.

**Results:**

Based on the system we calculate a sharpness measure and classify virtual slides into one of the following categories - excellent, okay, review and defective. Generating a scaled sharpness map enables the user to evaluate sharpness of WSIs and shows overall quality at a glance thus reducing tedious assessment work.

**Conclusions:**

Using sharpness assessment as an example, the introduced system can be used to process, analyze and parallelize analysis of whole slide images based on open source software.

## Background

Automated analysis of whole slide images (WSIs) is a growing challenge in scientific research in digital pathology. Image processing software usually works on standard-size images whereas virtual slides are very large. Due to the image size the computational cost of processing whole slide images in high resolution is immense. We present a system that combines virtual microscopy and public domain image analysis software running within a distributed parallel processing framework available in open source.

In terms of image analysis slide preparation, illumination, focus, and stitching are crucial factors for high quality scans. Image acquisition process for whole slide images has been considerably improved over the last years. Nevertheless, manual inspection of image sharpness is often still required after scanning. It is a tedious and time consuming task with a high inter and intra observer variability. We choose automatic sharpness assessment on histological WSIs as an exemplary application of distributed image analysis. It demonstrates how the new system can be used for standardized image quality assessment in high throughput scenarios.

## Methods

With the availability of public domain image processing libraries and free open source parallelization frameworks, we have combined these with recent virtual microscopy technologies such as WSI streaming servers [1,2] to provide a free processing environment for rapid prototyping of image analysis algorithms for WSIs.

NIH ImageJ [3,4] is an interactive open source image processing software and library developed at the National Institutes of Health. Its large user community contributes extensions (plug-ins) in a wide field of life science applications. Several plug-in bundles for microscopic images are available that are based on ImageJ [5,6]. We have developed a plug-in to add capabilities for handling WSIs.

Algorithms with large processing power requirements, such as most image analysis tasks on WSIs, can often be split into smaller parts and executed simultaneously on different computers. In this work the Java Parallel Processing Framework (JPPF) was used to process WSIs on standard PCs as compute nodes [7]. We employed an approach with non-overlapping tiles in a predefined resolution, where compute nodes retrieve image tiles of moderate size from a WSI streaming server. Each node processes the same set of ImageJ plug-ins independently. All resulting data are collected and merged within the JPPF environment using domain specific merger implementations.

Many different criteria exist to measure sharpness in an image [8-12]. Sun et al. compared 18 focus algorithms where sum-modified-Laplacian (SML) showed the best overall results [8]. Wei et al. confirmed its quality [9]. Besides, the well known Tenenbaum gradient has been found to be a reasonable focus measure when execution time is taken into account. Most publications deal with autofocus applications, where the sharpest image in a stack of focus images has to be found during image acquisition. In non z-stack WSIs sharpness has to be evaluated on a single focus plane after slide scanning. Analysis has to be done in full resolution, since sub sampling of images results in sharper images. We used a modified Tenenbaum gradient for sharpness assessment application and applied it to non-overlapping tiles. Compute nodes simultaneously determine the focus measure for distinct image tiles in full resolution. Each tile is divided into small sub images to calculate the sharpness measure. The results are aggregated in a sharpness map.

## Results

**Figure 1:**
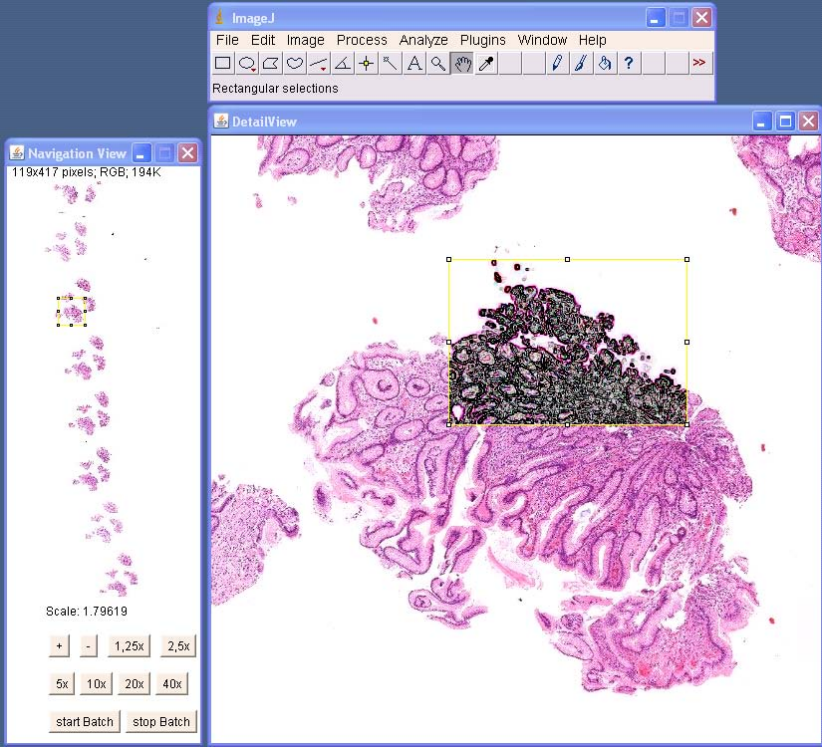
**ImageJ virtual microscope.** ImageJ was upgraded to a virtual microscope by connecting a multi format WSI streaming server as an image data provider.

We upgraded ImageJ to a virtual microscope (Fig. 1). Several WSI formats were added to ImageJ including JPEG2000 (ISO), NDPI (Hamamatsu), Zoomify (e.g. Metasystems, Leica), and Mirax (3DHISTECH). File system interfaces as well as web based WSI streaming servers are provided as image data sources to access virtual slides. Based on the ImageJ framework we developed a plug-in that shows a graphical user interface like a standard virtual microscope. Navigation elements such as panning and continuous zooming are offered. In addition to this, the microscope allows doing batch processing of WSIs.

**Figure 2:**
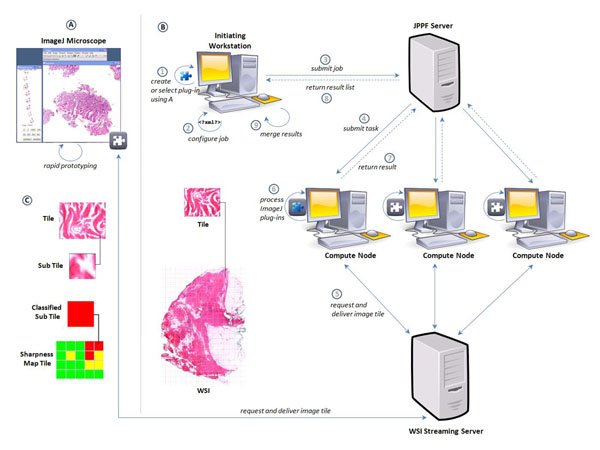
**System overview.** A) The ImageJ microscope can be used for rapid prototyping purposes. B) An image processing job is divided into several tasks. Image tiles are processed on a grid of standard PCs (compute nodes). Each node receives image tiles and processes predefined plug-ins. Results are merged using a domain specific implementation. C) Application to sharpness assessment.

We developed a seamless integration of the ImageJ plug-in interface and WSI streaming technologies within JPPF (Fig. 2). This enables the user to rapidly develop and test algorithms inside the ImageJ microscope and use this without any modifications inside a distributed and parallelized environment.

**Figure 3:**
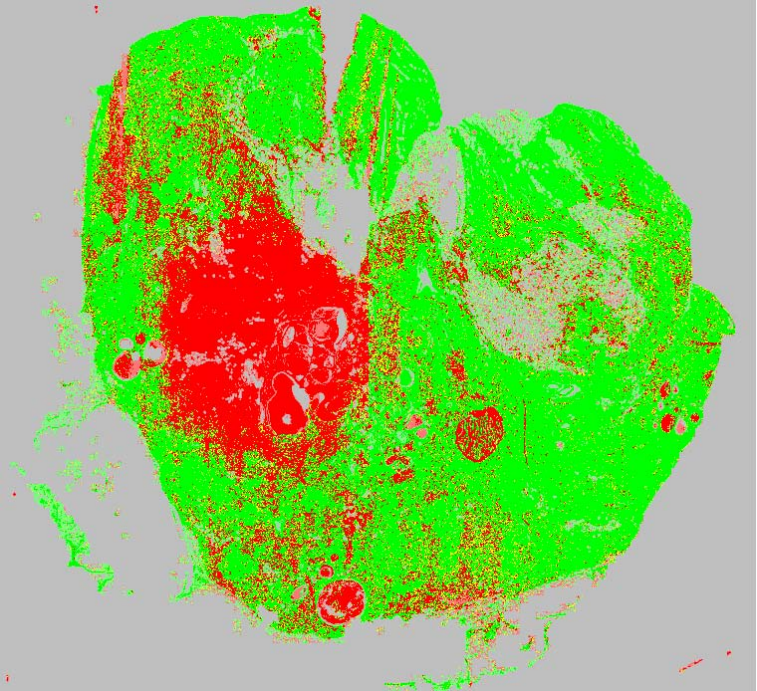
**Sharpness map.** Output of sharpness assessment procedure. Each pixel represents the result of a sub tile with a dimension of 128x128 pixels (green – sharp tile, red – blurred tile, light green – sharp tile with high percentage of background, light red – blurred tile with high percentage of background, yellow – medium sharp tile, gray – background).

The scalable system was employed to refine and parallelize a gradient based sharpness measure. Its results were used to generate a sharpness map for subsequent visual inspection (Fig. 3). The map allows a user to evaluate overall focus quality at a glance. Virtual slides are classified into four sharpness categories – excellent quality, acceptable quality, to be reviewed and defective quality.

**Figure 4:**
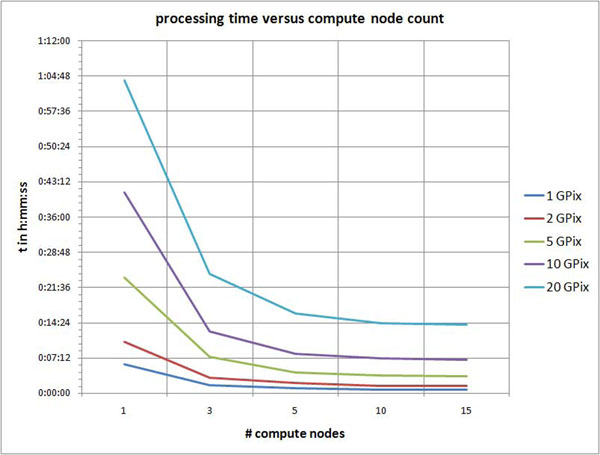
**Processing time.** Line chart of total processing time versus number of compute nodes and tissue area given in gigapixels. Time is measured in hours, minutes and seconds h:mm:ss.

Figure 4 shows the overall amount of processing time needed to inspect sharpness as a function of two variables, tissue area and number of compute nodes. Tissue area to inspect is given in gigapixels. Time is measured in hours, minutes and seconds h:mm:ss.

## Discussion

Automatic sharpness assessment of whole slide images is an exemplary application of the proposed framework. It could be shown that the chosen sharpness measure reproduces results of manual sharpness inspection.

JPPF offers functions to integrate standard PCs into a parallelization grid by running a worker node service with low priority. Standard office PCs can be employed for parallel processing purposes without interfering with regular work. Moreover, no additional costs for infrastructure or maintenance are generated.

So far limiting factors for scalability of the introduced system are 1) speed of delivering source images by the image provider and 2) amount of result data to be merged after processing. To improve image provider speed we are currently adding load balancing capabilities for multiple WSI streaming servers. Merging result data inside a relational database management system can resolve limitations of memory based merging.

## Conclusions

The most prominent toolkit in open source medical image analysis, ImageJ with its wide range of available algorithms, has been made applicable to virtual microscopy. Hence, based on the presented components it is possible to interactively as well as automatically process and analyze WSIs within ImageJ. Furthermore, distributed batch processing of WSIs using an open source parallel processing framework (JPPF) is seamlessly integrated with the plug-in concept of ImageJ.

The introduced open system architecture can be adapted within various application contexts and proves flexibility and scalability. Furthermore, it reduces processing time. The introduced bridging technology between ImageJ, JPPF, and virtual microscopy image streaming servers has applications in rapid prototyping of scientific image analysis software, education and is also an option for cost effective solutions in upcoming high throughput image analysis scenarios.

## Competing interests

The authors declare that they have no competing interests.
